# Role of CTCF in Regulating *SLC45A3-ELK4* Chimeric RNA

**DOI:** 10.1371/journal.pone.0150382

**Published:** 2016-03-03

**Authors:** Fujun Qin, Yansu Song, Yanmei Zhang, Loryn Facemire, Henry Frierson, Hui Li

**Affiliations:** 1 Department of Pathology, School of Medicine, University of Virginia, Charlottesville, Virginia, United States of America; 2 Zhejiang Academy of Medical Sciences, Hangzhou, Zhejiang, China; 3 Department of Biochemistry and Molecular Genetics, School of Medicine, University of Virginia, Charlottesville, Virginia, United States of America; University of Michigan, UNITED STATES

## Abstract

The chimeric RNA, *SLC45A3-ELK4*, was found to be a product of cis-splicing between the two adjacent genes (cis-SAGe). Despite the biological and clinical significance of *SLC45A3-ELK4*, its generating mechanism has not been elucidated. It was shown in one cell line that the binding of transcription factor CTCF to the insulators located at or near the gene boundaries, inversely correlates with the level of the chimera. To investigate the mechanism of such cis-SAGe events, we sequenced potential regions that may play a role in such transcriptional read-through. We could not detect mutations at the transcription termination site, insulator sites, splicing sites, or within CTCF itself in LNCaP cells, thus suggesting a “soft-wired” mechanism in regulating the cis-SAGe event. To investigate the role CTCF plays in regulating the chimeric RNA expression, we compared the levels of CTCF binding to the insulators in different cell lines, as well as clinical samples. Surprisingly, we did not find an inverse correlation between CTCF level, or its bindings to the insulators and *SLC45A3-ELK4* expression among different samples. However, in three prostate cancer cell lines, different environmental factors can cause the expression levels of the chimeric RNA to change, and these changes do inversely correlate with CTCF level, and/or its bindings to the insulators. We thus conclude that CTCF and its bindings to the insulators are not the primary reasons for differential *SLC45A3-ELK4* expression in different cell lines, or clinical cases. However, they are the likely mechanism for the same cells to respond to different environmental cues, in order to regulate the expression of *SLC45A3-ELK4* chimeric RNA. This response to different environmental cues is not general to other cis-SAGe events, as we only found one out of 16 newly identified chimeric RNAs showing a pattern similar to *SLC45A3-ELK4*.

## Introduction

Chimeric RNAs were thought to be generated solely by gene fusions at the DNA level. However, recent reports of chimeric RNAs joining adjacent genes suggest another mechanism. Various groups have named these chimeras “transcription-mediated gene fusions”, “tandem chimerism”, and “conjoined genes” [1–4]. We have used the phrase “cis-splicing between adjacent genes (cis-SAGe) to differentiate transcriptional read-through from trans-splicing [1, 5, 6]. Among the chimeras, *SLC45A3-ELK4* in prostate cancer was found by two groups independently [7, 8], and has recently gained attention because of its biomarker potential [1, 5, 7, 8]. The e4e2 form was originally discovered by RNA-Seq in prostate cancer, and it seems to be expressed at a higher level in a subset of prostate cancer samples than in benign prostate tissues [7]. Two recent studies also found this form of the fusion in normal margins, questioning its cancer specificty [9, 10]. However, it is not clear whether the fusion can be found in prostate tissues from non-cancer patients. The e1e2 form of *SLC45A3-ELK4* seems to correlates with prostate carcinogenesis [1, 7]. In addition, silencing the e1e2 form of the fusion RNA resulted in significant cell growth arrest in both androgen-dependent and castration-resistant prostate cancer cells [1]. Here, we focused on the e1e2 form. Despite the biological and clinical significance of this fusion RNA, its generating mechanism has not been elucidated. CCCTC-binding factor (CTCF) is a highly conserved zinc finger protein that plays a diverse role in regulatory functions, including transcriptional activation/repression, insulating, imprinting, and X chromosome inactivation [11]. Insulators between the neighboring genes act as boundaries to protect a gene against the encroachment of adjacent, inactive, condensed chromatin, or against the activating influence of distal enhancers associated with other genes [12]. Insulator activity is controlled mainly by CTCF as evidenced by enhancer blocking transgene assays [13], and genome-wide studies [14]. Previously, CTCF bindings to the two insulators at, or near the *SLC45A3* and *ELK4* gene boundaries were found to be negatively correlated with the expression of the fusion RNA [1]. Silencing CTCF also resulted in a higher expression of the chimera [1]. It was thus postulated that CTCF and its binding may be the key determining factor for the expression of the chimera. Surprisingly, in this study we found no supportive evidence for CTCF, or its bindings to the insulators to explain the difference of the fusion RNA level among different cell lines and clinical samples. We carefully examined the androgen and CTCF effect on the fusion in LNCaP cells, and confirmed that androgen treatment lead to decreased CTCF binding to the insulator, and increased the fusion RNA expression. We then examined two castration-resistant lines, and found that with serum, CTCF and its bindings to the insulators were reduced. At the same time, the fusion RNA was induced. These results are consistent with the model that CTCF regulates the expression of the fusion RNA under different environmental conditions for the same cells, but is not the key factor determining the expression of fusion RNA in different cells. This response to androgen and serum is not universal, as we found only one more such fusion in 16 other cis-SAGe fusion RNAs that responded similarly.

## Materials and Methods

### Clinical samples

The use of the human clinical samples was approved by the IRB committee of the University of Virginia. The IRB committee has waived the need for patient consent. All the samples were de-identified.

### Cell culture and antibodies

LNCaP and PC-3 cells were grown in RPMI1640 (Hyclone) media supplemented with 10% FBS and 1% Pen/Strep solution (Hyclone). HCT116, Hela, A2780, and 293T cells were maintained in DMEM (Hyclone) media supplemented with 10% FBS and 1% Pen/Strep solution (Hyclone). For androgen treatment, cells were hormone starved for 2–3 days in RPMI 1640 (Phenol free) media, supplemented with 5% charcoal-striped FBS, and treated with 1 nM R1881 for 24 h. Antibodies including anti-AR (06–680) from Millipore and anti-CTCF (3418) from Cell Signaling were used in this study.

### RNA extraction and Reverse Transcription

RNA was extracted from related cell lines using Trizol (Life Technology), following the manufacturer’s instruction. Clinical tissue samples were pulverized under liquid nitrogen, and RNA was extracted using Trizol reagent. All of the RNA samples used in this study were treated with DNase I, followed by standard Reverse Transcription using AMV RT (NEB).

### Chromatin immunoprecipitation (ChIP)

CTCF ChIP was carried out as described previously with a slight modification [15, 16]. Briefly, cultured cells (5x10^6^ cells each ChIP), or tissue cells were resuspended in 1X PBS, cross-linked with 1% formaldehyde for ten minutes, followed by cross-linking inactivation using 0.125 M glycine for five minutes at room temperature with gentle shaking. Cells were then rinsed twice with cold 1X PBS. The following steps were performed at 4 °C. Cell pellets were resuspended and incubated in cell lysis buffer with 10 ul ml^−1^ PMSF and protease inhibitor (Roche) for ten minutes. Nuclei pellets were spun down at 3,000 *rpm* for five minutes, resuspended in nuclear lysis buffer, and then incubated for another ten minutes on ice. Chromatin was sonicated to an average length of 500 bp, and then centrifuged at 13,000 *g* for 15 minutes to remove debris. Supernatants containing chromatin fragments were incubated with agarose/protein A, or beads (Millipore) for 15 minutes, and centrifuged at 1,000 *rpm* for three  minutes to reduce nonspecific binding. Five percent of pre-cleared chromatin was used as input. To immunoprecipitate protein/chromatin complexes, the supernatants were incubated with 1 ug of antibody (anti-CTCF from Cell Signaling, cat# 3418) overnight, then 50 ul of agarose/protein A or G beads were added and incubated for two hours. Beads were washed twice with low salt buffer, high salt buffer, LiCl buffer, and TE buffer, respectively. The antibody/protein/DNA complexes were eluted twice with 250 ul IP elution buffer. To reverse the cross-links, the complexes were incubated in elution buffer with 20ul 5M NaCl at 67 °C overnight. DNA/proteins were incubated for two hours at 45°C, with 10ul of 0.5M EDTA, 20ul Tris-HCl (1M pH 6.5), 2ul of 10 mg/ml proteinase K treatment, and 5ul of 10mg/ml RNase. The DNA/protein complexes were precipitated with ethanol, air-dried, and dissolved in 30 ul of TE. The subsequent ChIP quantitative real-time PCR was used to measure the enrichment of the target gene region. The enrichment analysis was performed by Comparative Ct method, and normalized to the input, that is, enrichment over input = 2^(−ΔCt)^, where ΔCt = Ct sample−Ct input [16, 17].

### Re-Chromatin immunoprecipitation (Re-ChIP)

CTCF and AR Re-ChIP was modified from the previously described [18]. Re-ChIP assays follow the ChIP protocol described above. Following the initial overnight immunoprecipitation, protein-DNA-bead complexes were washed three times with Re-ChIP wash buffer, followed by a double wash with 1x TE buffer. The complexes were eluted with 75 ul Re-ChIP elution buffer. Protein-DNA-bead complexes were diluted 20 times (to a final volume of 1.5ml) with ChIP dilution buffer supplemented with 50ug BSA and protease inhibitor (Roche). The second immunoprecipitation reaction, and rest of the procedure was performed following the ChIP protocol above.

### Quantitative Reverse Transcription Polymerase Chain Reaction (qRT-PCR)

All primers in this study (S1 Table) were designed using Primer 3 (http://frodo.wi.mit.edu/primer3/), and synthesized by Eurofins MWG Operon. SYBR Green based qRT-PCR was performed using ABsolute Blue qPCR SYBR Green ROX Mix (AB-4162, Thermo Scientific), using a Step One Plus Real-Time PCR System (Applied Biosystems). For qRT-PCR data analysis, the fold change in the target gene relative to the GAPDH (glyceraldehyde 3-phosphate dehydrogenase) endogenous control gene was determined by the 2^–Δ(ΔCt)^ method, as described previously [6, 15, 18–21].

## Results

### No point mutations were found responsible for the formation of *SLC45A3-ELK4*

**Fig 1:**
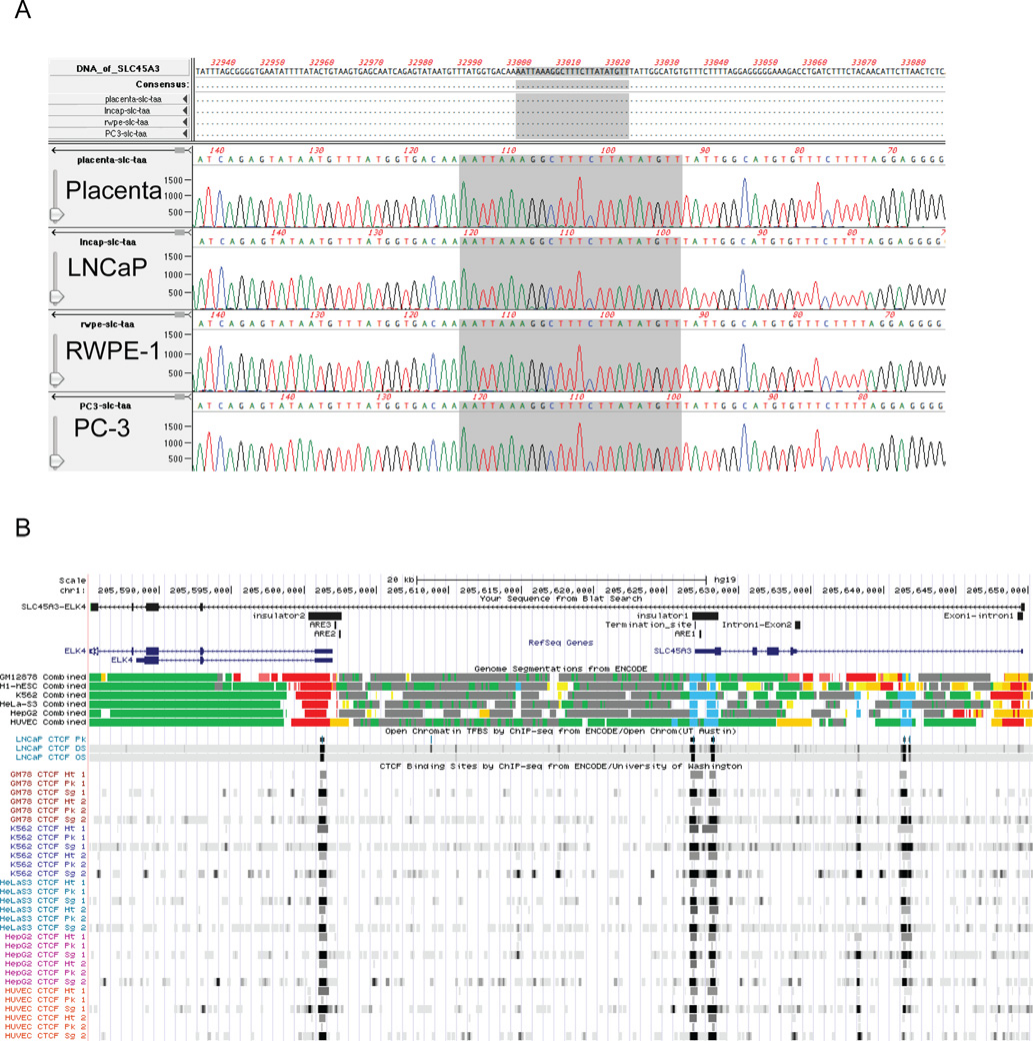
**No Mutation around *SLC45A3* gene poly (A) signal, termination site, splicing sites, and insulator regions in LNCaP cells.** (A) Sequence alignment of poly (A) signal in LNCaP, PC3, RWPE-1, and a normal placenta. No mutation was observed. (B) UCSC genome browser screenshot of the regions of interest including insulators, AREs, termination site, *SLC45A3-ELK4* e1e2 form fusion RNA, Genome Segmentation characterization by ChromHMM, and CTCF ChIP-Seq mapping.

We reasoned that cis-SAGe requires the primary transcript to not stop at the termination site, and to run through the gene boundaries. Mutation at the poly (A) signal has been reported to disrupt the normal site of transcriptional termination, and blocks mRNA polyadenylation [22]. In mild thalassemia, a point mutation in the canonical poly (A) signal (AAUAAA) of the *HBB* gene causes a larger transcript to be produced [23]. To investigate whether any point mutations at the poly (A) site could explain the continuation of the *SLC45A3* transcript, we sequenced the last exon of the *SLC45A3* gene in LNCaP (a prostate cancer cell line responsive to androgen), PC3 (a prostate cancer cell line unresponsive to androgen), RWPE-1 (a benign prostate epithelial cell line), and a normal placenta control. No mutation was found at the predicted poly (A) signal (in this case AUUAAA), or in the G/U rich region downstream (Fig 1A), even though both LNCaP and PC3 express high levels of *SLC45A3-ELK4* chimeric RNA [1]. Cis-SAGe is in essence an alternative splicing between neighboring genes. To rule out the abnormalities at, or near the splicing sites of the *SLC45A3* gene, we performed Sanger sequencing for the exon1-intron1 and intron1-exon2 regions (Fig 1B), including splicing donor and acceptor sites, and branch sites. No mutations were observed in at least the 300bp regions flanking the splicing sites among LNCaP, PC3, RWPE-1, and the placenta control, except a single nucleotide polymorphism (SNP) (S1 Fig).

Previously, we reported an inverse correlation between *SLC45A3-ELK4* expression and CTCF binding to the insulators at or near the gene boundaries in LNCaP cells [1]. When we sequenced the insulators (Fig 1B) in LNCaP cells, no abnormality was found. We also sequenced the CTCF coding region in LNCaP cells, and found no mutation (data not shown).

These results suggest that instead of “hard-wired” nucleic acid mutations at DNA level, some “soft-wired” mechanism may control the formation of *SLC45A3-ELK4*.

### Absence of the inverse correlation between CTCF binding to the insulator regions and the chimera expression among different cell lines

**Fig 2:**
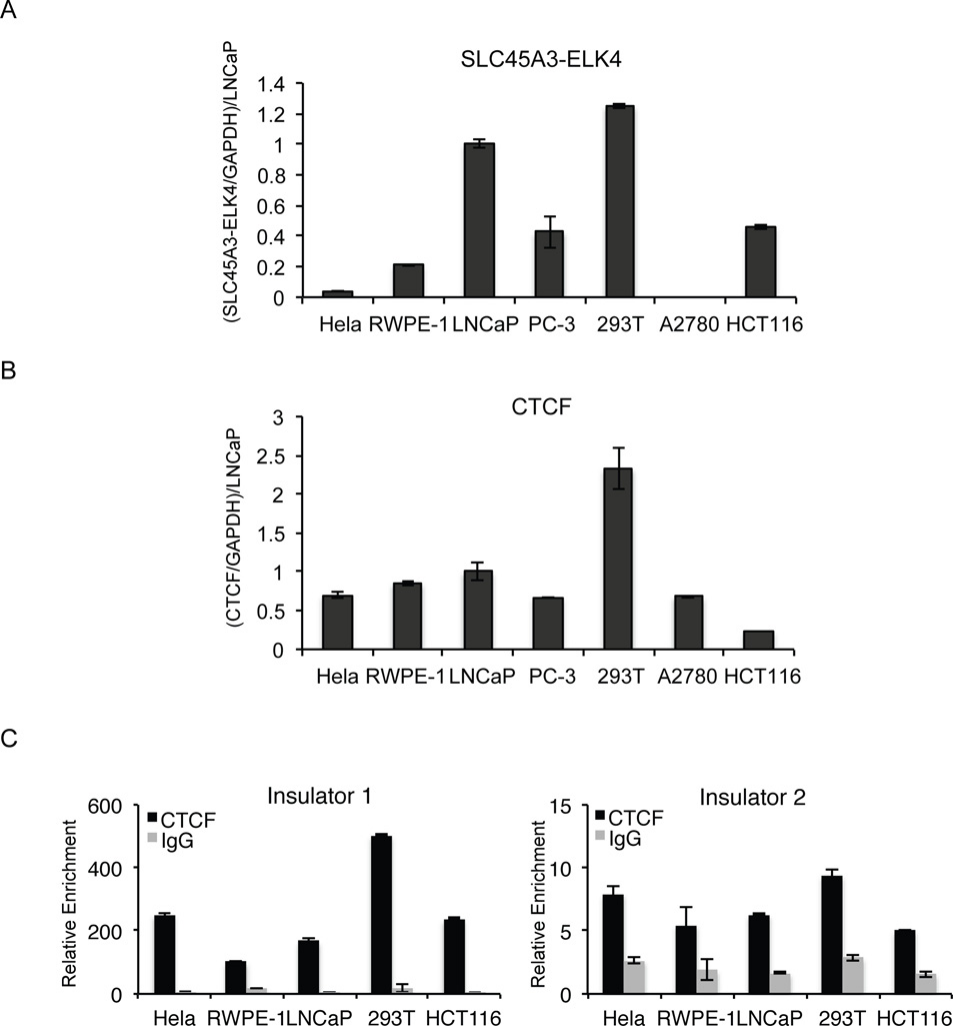
Expression of *SLC45A3-ELK4* and *CTCF*, and CTCF binding to the two insulators in different cell lines. Hela (cervical cancer cells), 293T (SV40 transformed embryonic kidney cells), HCT116 (colon cancer cells), LNCaP, and RWPE-1 (prostate cells). (A) *SLC45A3-ELK4* expression level was measured by qRT-PCR, normalized against *GAPDH*, and further normalized against the level in LNCaP. (B) *CTCF* expression level measured by qRT-PCR, normalized against *GAPDH*, and further normalized against the level in LNCaP. (C) Binding of CTCF to the two insulators measured by ChIP and qPCR. IgG was used as control for the CTCF antibody.

We then tested whether CTCF and its bindings to the insulators located at, or near the two gene boundaries would inversely correlate with *SLC45A3-ELK4* expression among different cells. In addition to prostate cancer cells, we could detect *SLC45A3-ELK4* in other cancer cell lines. We noticed a higher level of *SLC45A3-ELK4* in 293T, LNCaP, PC3, and HCT116 (Fig 2A). However, CTCF expression is also high in 293T and LNCaP cells (Fig 2B). In fact, there is even a positive correlation (S2A Fig). We then performed ChIP to measure the CTCF binding to the insulators we previously reported [1] (positive control for CTCF binding is shown in S2B Fig). We did not observe an inverse correlation between the CTCF binding and *SLC45A3-ELK4* expression (Fig 2C). 293T cells have a higher level of CTCF binding than other cell lines, but they also have the highest level of the chimeric RNA expression (S2A Fig).

### Lack of inverse correlation in clinical samples

**Fig 3:**
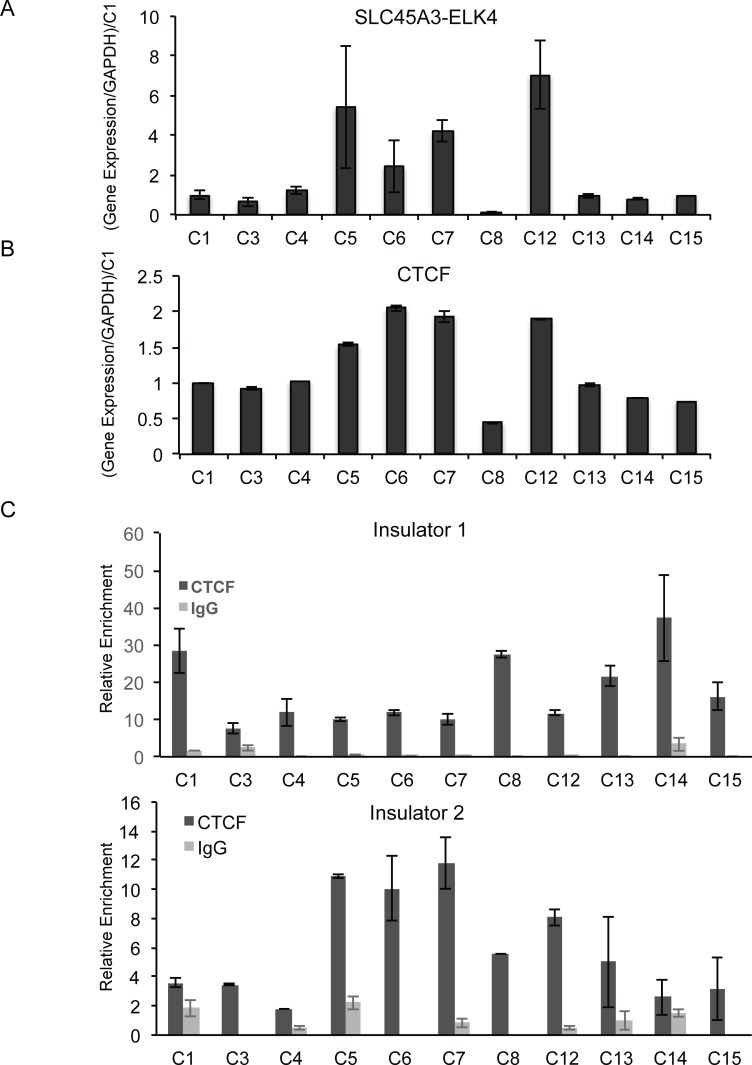
Expression of *SLC45A3-ELK4* and *CTCF*, and CTCF binding to the two insulators in 11 clinical prostate cancer samples. (A) *SLC45A3-ELK4* expression level was measured by qRT-PCR, normalized against *GAPDH*, and further normalized against the level in C1. (B) *CTCF* expression level measured by qRT-PCR, normalized against *GAPDH*, and further normalized against the level in C1. (C) Binding of CTCF to the two insulators measured by ChIP and qPCR. IgG was used as control for the CTCF antibody.

The comparison above involves multiple cancer types, and is limited to just cell lines. To focus the study in prostate cancer, and to investigate the situation in clinical samples, we acquired 11 frozen prostate cancer biopsies (S2 Table). We measured *SLC45A3-ELK4* (Fig 3A) and CTCF (Fig 3B) levels with qRT-PCR, and found not an inverse, but a positive correlation (S3A Fig). In this collection, we also did not observe an obvious correlation of Gleason score with the level of *SLC45A3-ELK4*. We then tested whether a reliable ChIP can be performed with these frozen clinical samples. The samples were pulverized within liquid nitrogen, and were re-suspended in 1X PBS. Standard ChIP protocol was followed. Significant signal above IgG background was seen at the HS5 locus, a positive control for CTCF binding (S3B Fig). Using this protocol, we detected strong CTCF signal at insulator1, and weaker, but still above background signal at insulator 2 (Fig 3C). However, no obvious inverse correlation, even with a light positive correlation for insulator2, was seen between CTCF binding and *SLC45A3-ELK4* expression (S4A and S4B Fig).

### Fluctuation of *SLC45A3-ELK4* under different growth conditions inversely correlates with CTCF binding to the insulator(s)

**Fig 4:**
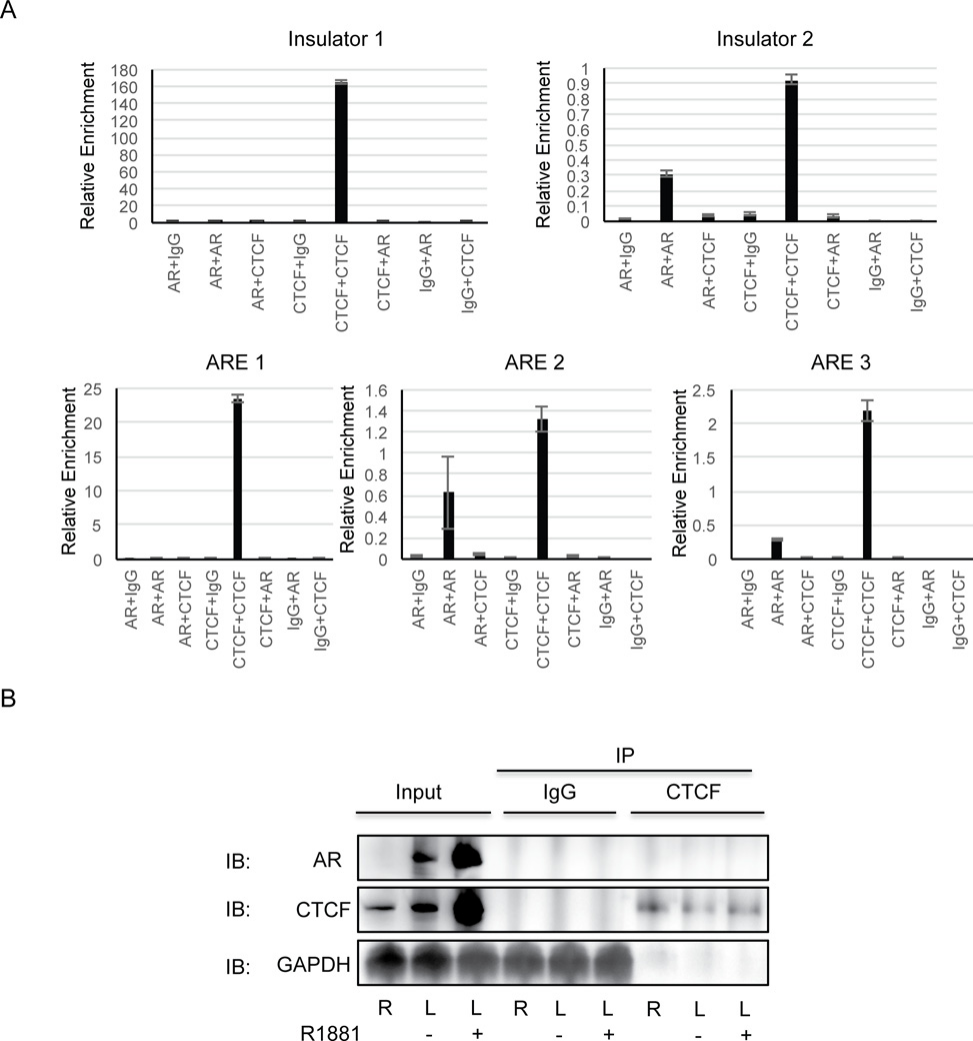
AR and CTCF do not colocalize, or interact in LNCaP cells. (A) Re-ChIP by CTCF, AR antibodies, or control IgG. Insulator1, 2 and, ARE1, 2, and 3 were tested. (B) Co-immunoprecipitation by CTCF antibody, and western blotting by AR, CTCF, or GAPDH. R-RWPE-1, L-LNCaP, R1881-synthetic androgen.

At first glance, the above results seem to contradict our original observation in LNCaP cells, that CTCF binding to the insulators negatively correlates with the fusion RNA expression [1]. We then re-examined the CTCF bindings at the insulators with or without androgen, and found again that the induction of *SLC45A3-ELK4* correlates with a reduction of CTCF binding to the insulators (S5 Fig). We then hypothesized that AR competes with CTCF based on the following observations: 1). the level of *SLC45A3-ELK* is increased when treated with synthetic androgen [1]; 2). the level of *SLC45A3-ELK4* in charcoal-stripped FBS medium (no androgen) is much lower than in regular FBS medium (containing androgen) [1]; 3). CTCF bindings to the insulator were reduced by synthetic androgen (S5 Fig); 4). knocking down of CTCF increased *SLC45A3-ELK4* expression level [1]. To gain more support for the competition model, we measured androgen receptor (AR) and CTCF binding to the insulators. Re-ChIP (scheme in S6 Fig) with sequential AR and CTCF antibody pull-down found no evidence of AR and CTCF colocalizing at either insulator (Fig 4A). Consistently, no signal of colocalization was seen at any of the three ARE sites (Fig 4A). Supporting the idea that AR and CTCF might compete for the binding region, we found that both CTCF and AR bind to insulator2, and ARE2 and ARE3. We also performed protein co-immunoprecipitation, and saw no evidence of interaction between the two proteins in RWPE-1, or in LNCaP cells even with androgen (Fig 4B). These findings give possible credence to the idea that in the presence of androgen, AR might compete with CTCF for the binding around the insulators, and induce the expression of the chimeric RNA.

**Fig 5:**
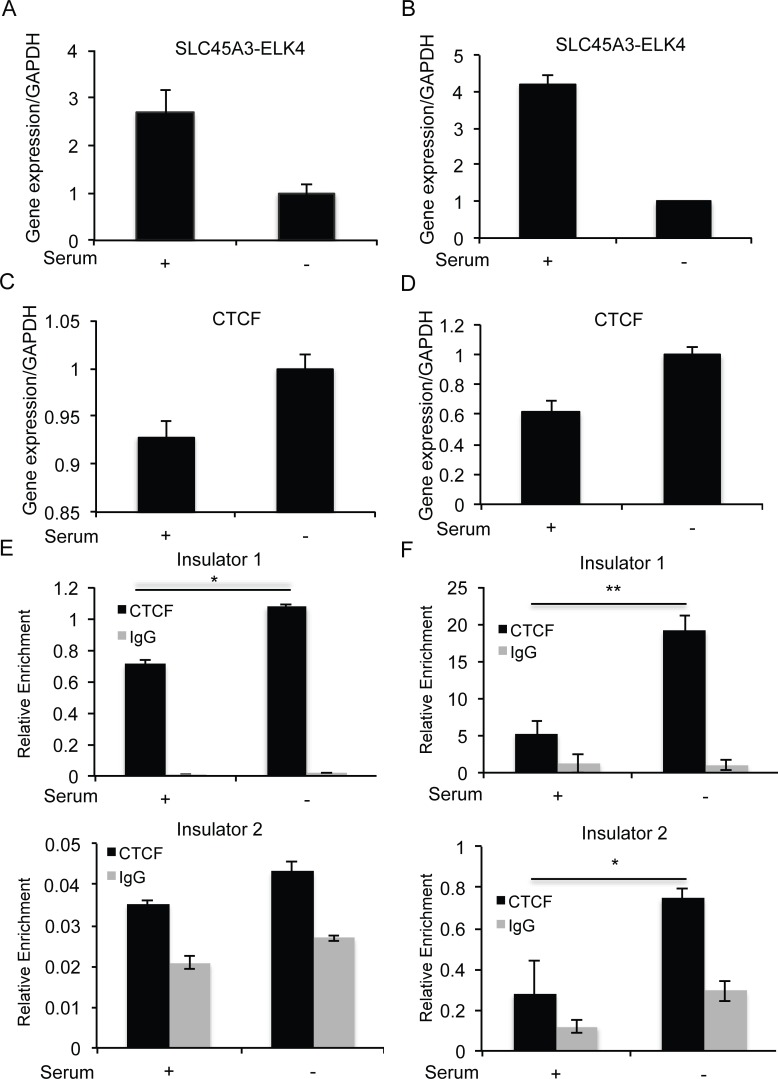
**In the situation of castration-resistant prostate cancer cells, C4-2 (A, C, E) and PC3 (B, D, F), serum induced the expression of *SLC45A3-ELK4*, reduced *CTCF* expression and reduced CTCF binding to the insulator(s).** (A) (B) *SLC45A3-ELK4* expression level was measured by qRT-PCR, normalized against *GAPDH*, and further normalized against the level in “Serum-”. (C) (D), *CTCF* expression level measured by qRT-PCR, normalized against *GAPDH*, and further normalized against the level in “Serum-”. (E) (F), Binding of CTCF to the two insulators measured by ChIP and qPCR. IgG was used as negative control for the CTCF antibody. * p<0.05, **p<0.01.

To extend our observation, and to further support the hypothesis that different environmental cues might trigger differences in CTCF binding to the insulators, and regulate *SLC45A3-ELK4* expression, we performed the study in two additional prostate cancer cell lines. Different from LNCaP, C4-2 and PC3 do not respond to androgen. Instead of synthetic androgen, we used serum as the stimuli. We noticed that *SLC45A3-ELK4* was induced in the presence of serum (Fig 5A and 5B). In contrast, CTCF level was reduced in both cell lines (Fig 5C and 5D). CTCF binding to both insulators was also reduced in PC3 (Fig 5E). In C4-2, CTCF binding to insulator1, but not insulator2, was significantly reduced in the presence of serum (Fig 5F).

**Fig 6:**
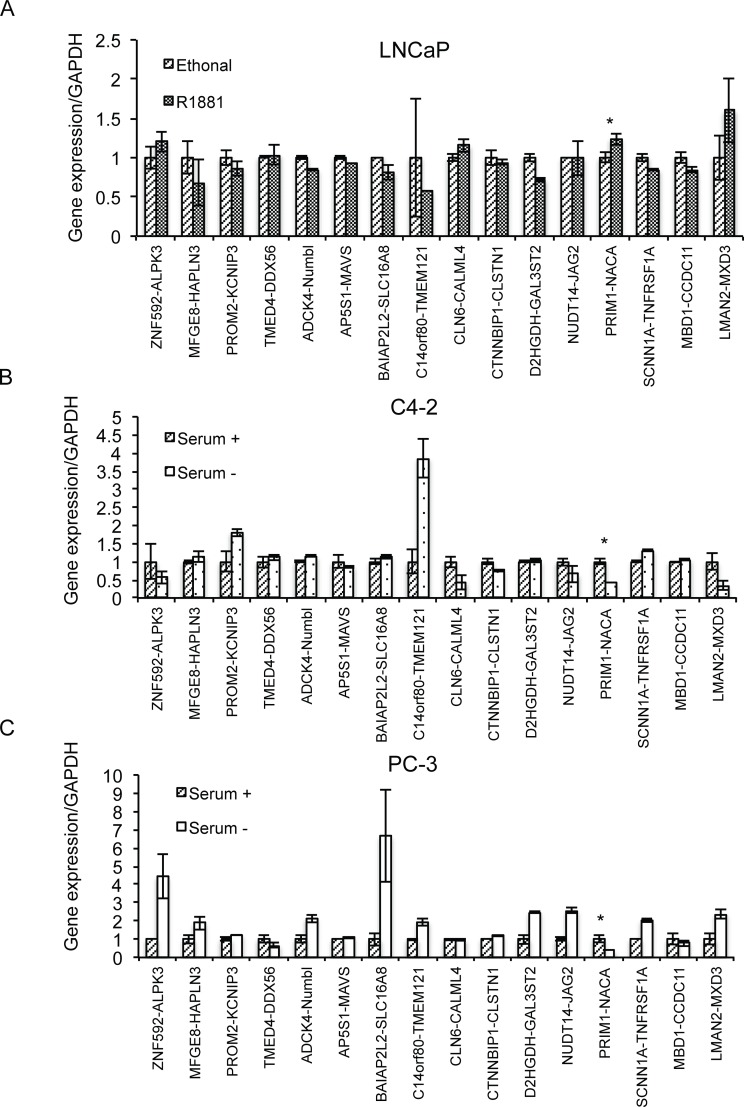
16 other cis-SAGe fusion RNAs in response to androgen in LNCaP cells, and serum in PC3 and C4-2 cells. (A) In LNCaP cells with or without androgen, fusion RNAs were measured by qRT-PCR, and normalized against *GAPDH*. In C4-2 cells (B) and PC3 cells (C) with or without serum, fusion RNAs were measured by qRT-PCR, and normalized against *GAPDH*.

We then investigated whether the same environmental cues would regulate other cis-SAGe fusions. We recently discovered an additional 16 new cis-SAGe fusions in LNCaP cells [6]. When LNCaP cells were exposed to androgen, only one fusion, *PRIM1-NACA* was significantly induced (Fig 6A). Interestingly, the same fusion was also induced with serum in C4-2 and PC3 cells (Fig 6B). These results suggest that not all cis-SAGe fusions respond to the same environmental cues, and that *PRIM1-NACA* may be regulated similarly to *SLC45A3-ELK4*.

## Discussion

Different from traditional chimeric RNAs, *SLC45A3-ELK4* is a fusion RNA generated by transcription readthrough/cis-SAGe. We believe that CTCF and its binding to the insulator regions at or near the gene boundaries negatively impact this cis-splicing between the two neighboring genes. The original speculation was that we should observe an inverse correlation between CTCF binding to the insulators and *SLC45A3-ELK4* expression in different samples. However, we failed to observe such an inverse correlation among different cell lines, or clinical samples. We even observed some positive correlation between CTCF expression level and the fusion RNA, even though the higher levels of CTCF expression did not translated into higher levels of CTCF binding to the insulators. These findings, even though contradicting to our original thinking, is important, as both the chimeric RNA and CTCF have recently attracted significant amounts of attention. Interestingly, in three prostate cancer cell lines, when cells were cultured under different conditions, we did observe the inverse correlation. With androgen (in LNCaP) or serum (in C4-2 and PC3), CTCF and/or its binding to the insulators were reduced, and *SLC45A3-ELK4* expression was induced. Altogether, these results suggest that the level of CTCF binding to the insulators are not the most prominent factors determining the level of this cis-SAGe among different cells. However, it may be an adaptive strategy to respond to different environmental cues, and the changes of CTCF expression, and/or its binding to the insulators may facilitate the production of the chimeric RNA. Androgen signaling is one of the most important pathways in prostate cancer tumorigenesis. Castration-resistant prostate cancers still use circuitous means to activate androgen signaling. Interestingly, a higher level of AR expression was shown to correlate with higher Gleason. It is thus not unlikely that AR, or AR signaling axis is the determining factor for *SLC45A3-ELK4*. To identify the exact factor in the serum that induces the changes in castration-resistant cells still requires further work.

We consider that cis-SAGe is essentially alternative splicing between neighboring genes. For it to happen, the primary transcript needs to travel through the gene boundary. Insulators between the neighboring genes act as boundaries to protect a gene against the encroachment of adjacent, inactive condensed chromatin, or against the activating influence of distal enhancers associated with other genes [12]. Insulator activity is controlled mainly by CTCF, as evident by enhancer blocking transgene assays [13], and genome-wide studies [14]. CTCF has also been shown to play a role in transcription termination [24]. However, our data suggest that it is not the key, rate-limiting factor that explains the variation in the expression level of the chimeric RNA, *SLC45A3-ELK4*, among different cell lines or clinical samples. In addition, CTCF has roles other than transcriptional insulation. The insulator2 is located near the promoter region of *ELK4* based on the Genome segmentation by ChromHMM from ENCODE. This could be the reason that CTCF has a lower binding affinity to the insulator2.

The absence of mutations at the poly (A) signal, insulators, splicing sites, and CTCF coding region hints on some “soft-wired” dynamics regulation of the fusion formation, rather than “hard-wired” mutations at the DNA level. The changes in CTCF, and/or its binding to the insulator regions under different growing conditions further support the model that cells respond to environmental cues by adjusting CTCF activity to regulate the level of chimeric RNA, *SLC45A3-ELK4*.

## Supporting Information

S1 Fig Sanger sequencing of the regions near the splicing sites of *SLC45A3* in LNCaP, PC3, RWPE-1, and the placenta control. (A) Sequence alignment for the region flanking the splicing donor site of exon1. Red arrow points to the SNP rs1772137. (B) Sequence alignment for the region flanking the splicing acceptor site of exon2.
(TIFF)

S2 Fig **(A) In different cell lines, instead of an inverse correlation, CTCF level seems to correlate with *SLC45A3-ELK4* level. (B) HS5 region was used as positive control for CTCF ChIP in various cell lines.** The binding of CTCF was measured by qPCR. IgG was used as negative control.
(TIFF)

S3 Fig **(A) In clinical samples of prostate cancer, instead of an inverse correlation, CTCF level seems to correlate with *SLC45A3-ELK4* level. (B) HS5 region was used as positive control for CTCF ChIP in frozen clinical samples.** The binding of CTCF was measured by qPCR. IgG was used as negative control.
(TIFF)

S4 Fig Absence of inverse correlation of CTCF binding to the insulators with *SLC45A3-ELK4* expression in 11 clinical prostate cancer samples. (A) Insulator1. (B) Insulator2.
(TIFF)

S5 Fig Response of *SLC45A3-ELK4* expression and CTCF binding to the two insulators when LNCaP cells were treated with androgen. (A) *SLC45A3-ELK4* expression level was measured by qRT-PCR, normalized against *GAPDH*, and further normalized against the level with no androgen. (B) Binding of CTCF to the two insulators measured by ChIP and qPCR. IgG was used as control for the CTCF antibody. * p<0.05, **p<0.01.
(TIFF)

S6 Fig Scheme for Re-ChIP. (TIFF)

S1 Table Primers used for PCR, ChIP-PCR and qRT-PCR. (XLSX)

S2 Table Gleason score and stage of the clinical samples. (XLS)
