# Supplementary figures and images for: Role of CTCF in Regulating SLC45A3-ELK4 Chimeric RNA
Source: PLoS One. 2016 Mar 3;11(3):e0150382. doi: 10.1371/journal.pone.0150382 (PMC4777538; doi:10.1371/journal.pone.0150382)

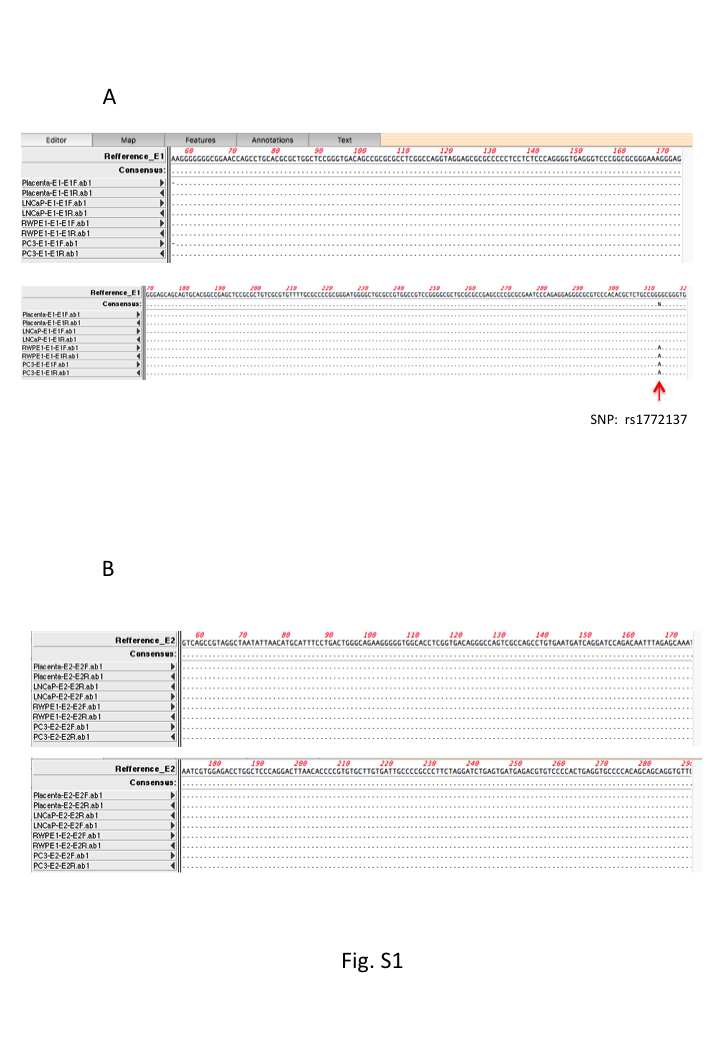

Supplement: S1 Fig — (A) Sequence alignment for the region flanking the splicing donor site of exon1. Red arrow points to the SNP rs1772137. (B) Sequence alignment for the region flanking the splicing acceptor site of exon2. (TIFF) [file pone.0150382.s001.tiff]

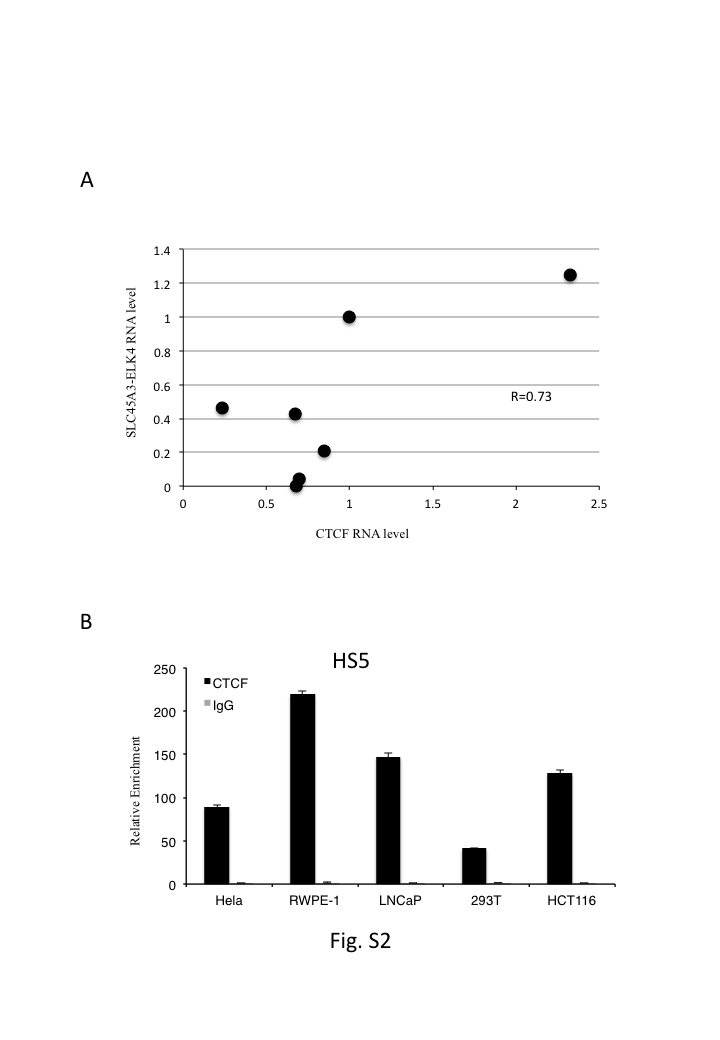

Supplement: S2 Fig — (A) In different cell lines, instead of an inverse correlation, CTCF level seems to correlate with SLC45A3-ELK4 level. (B) HS5 region was used as positive control for CTCF ChIP in various cell lines. The binding of CTCF was measured by qPCR. IgG was used as negative control. (TIFF) [file pone.0150382.s002.tiff]

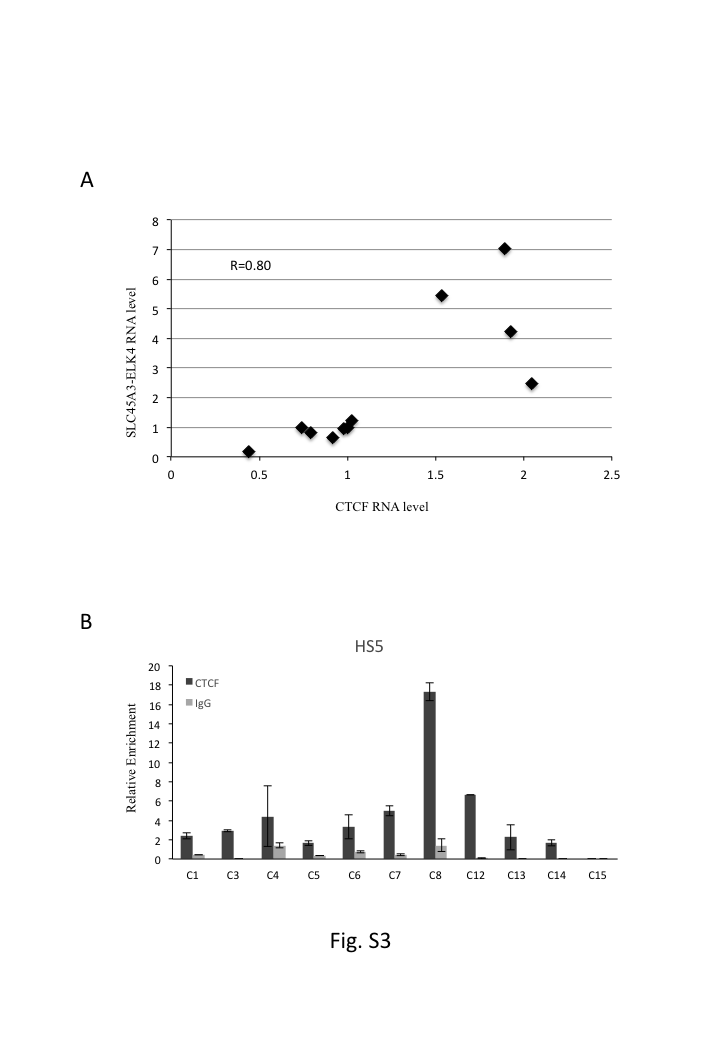

Supplement: S3 Fig — (A) In clinical samples of prostate cancer, instead of an inverse correlation, CTCF level seems to correlate with SLC45A3-ELK4 level. (B) HS5 region was used as positive control for CTCF ChIP in frozen clinical samples. The binding of CTCF was measured by qPCR. IgG was used as negative control. (TIFF) [file pone.0150382.s003.tiff]

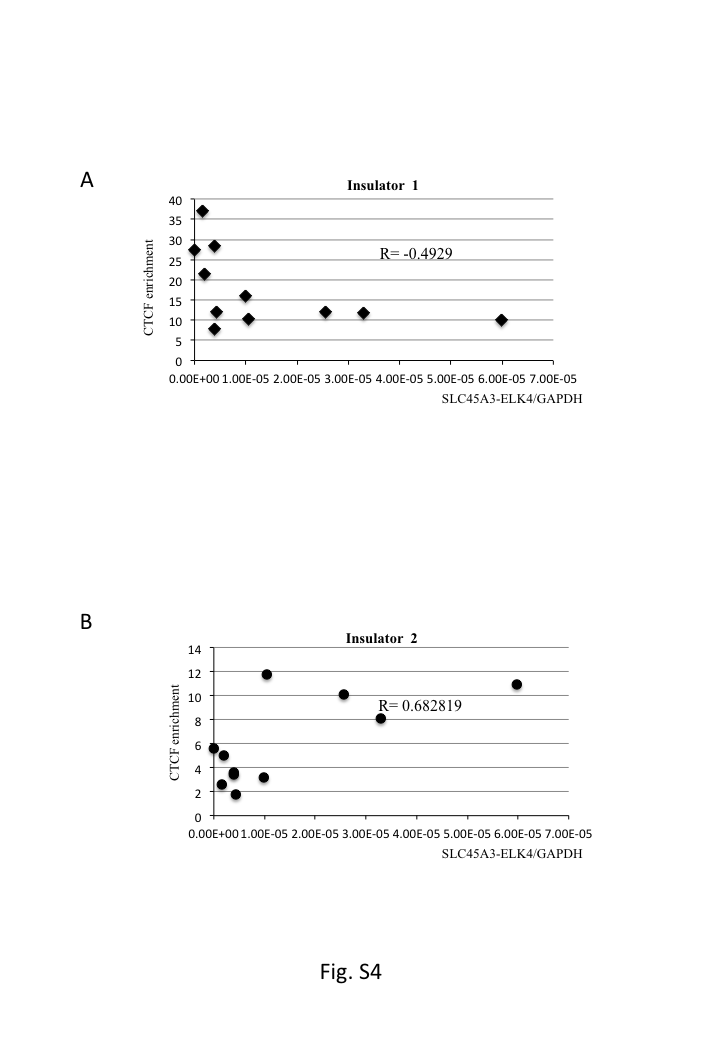

Supplement: S4 Fig — (A) Insulator1. (B) Insulator2. (TIFF) [file pone.0150382.s004.tiff]

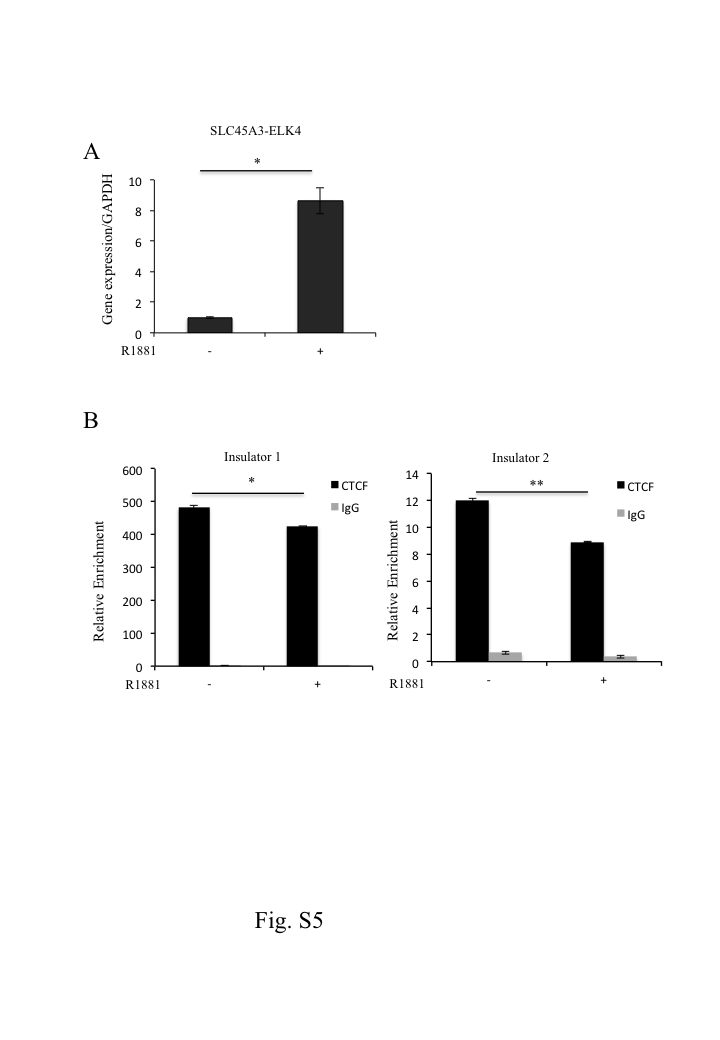

Supplement: S5 Fig — (A) SLC45A3-ELK4 expression level was measured by qRT-PCR, normalized against GAPDH, and further normalized against the level with no androgen. (B) Binding of CTCF to the two insulators measured by ChIP and qPCR. IgG was used as control for the CTCF antibody. * p<0.05, **p<0.01. (TIFF) [file pone.0150382.s005.tiff]

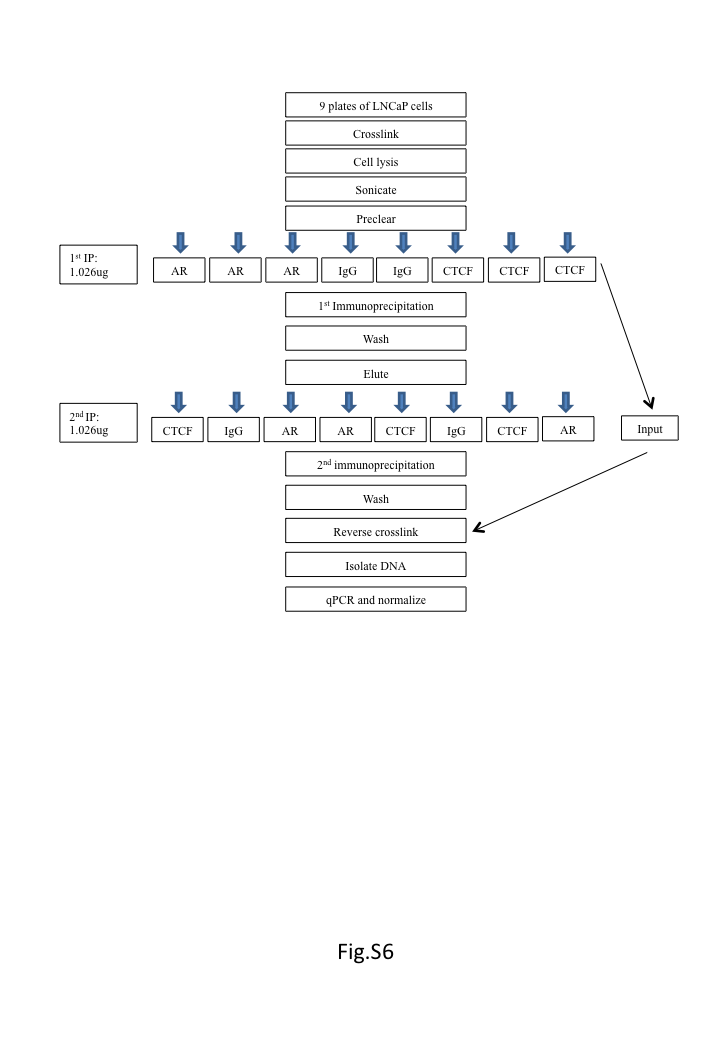

Supplement: S6 Fig — (TIFF) [file pone.0150382.s006.tiff]
